# Paracoccidioidomycosis: An atypical presentation in an immunocompetent woman

**DOI:** 10.1016/j.jdcr.2023.06.039

**Published:** 2023-07-08

**Authors:** Marita Yaghi, Alexandra C. Gamret, Scott Elman, Paolo Romanelli, Jonette E. Keri

**Affiliations:** a Dr. Phillip Frost Department of Dermatology and Cutaneous Surgery, University of Miami Miller School of Medicine, Miami, Florida; b Dermatology Service, Miami VA Hospital, Miami, Florida

**Keywords:** immunocompetent, infectious, mycoses, paracoccidioidomycosis, skin diseases, tropical medicine

## Introduction

Paracoccidioidomycosis (PCM) is a systemic mycotic disease endemic to Central and South America with the highest prevalence in Brazil, Argentina, and Colombia.^1^ Exposure to the dimorphic fungus conidia in infected soil in rural areas, often through coffee, cotton, or tobacco farming, induces human disease,^2^ which classically manifests with systemic symptoms, lymphadenopathy, and eosinophilia. Acute juvenile PCM typically manifests with skin and reticuloendothelial system involvement. In contrast, chronic adult PCM predominantly causes pulmonary and orofacial mucocutaneous disease.^3^ Papules, plaques, and nodules with varying ulceration and verrucous appearance are the hallmark of cutaneous involvement, most frequently affecting immunosuppressed patients.^3^

Here, we present a case of an immunocompetent woman with an unconventional presentation of disseminated PCM with extensive cutaneous involvement characterized by atypical skin lesions.

## Case presentation

A 27-year-old woman presented to the clinic with progressively worsening painful cystic nodules on her face for the past 2 months, unresponsive to a 5-week course of doxycycline. She reported a 25-pound unintentional weight loss over the past 6 months with accompanying fatigue, chills, night sweats, shortness of breath, and diarrhea. She moved from Brazil to Florida 2 years prior. Previous work-up by an outside dermatologist, rheumatologist, and hematologist was unrevealing despite a skin biopsy, bacterial and fungal cultures, and malignancy screening. Significant lymphadenopathy was also present and lymph node aspirate showed cystic follicular infundibular hyperplasia and hyperkeratosis, with granulomatous response and yeast forms suggestive of Cryptococcus neoformans.

**Fig 1:**
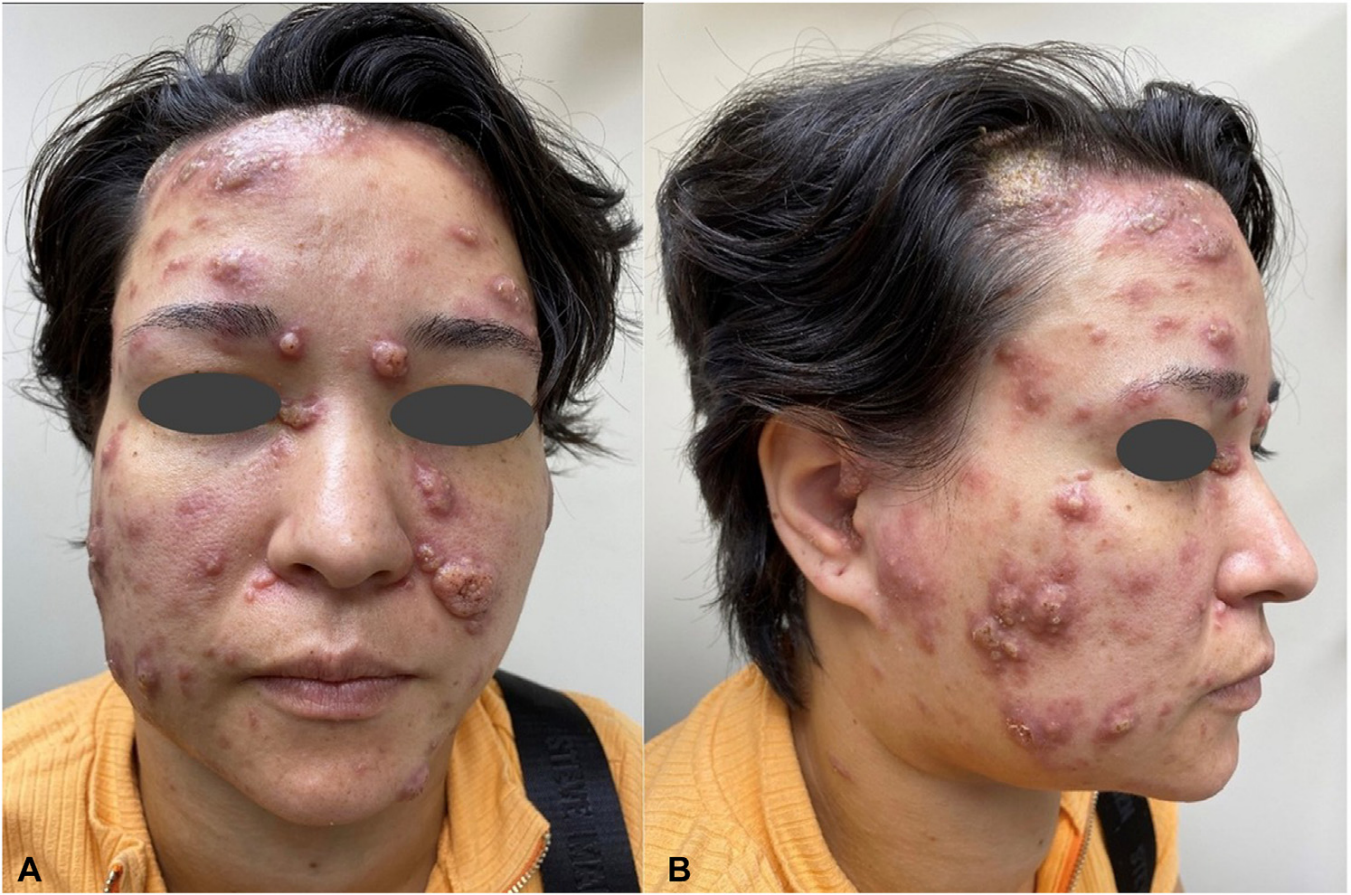
Systemic paracoccidioidomycosis cutaneous manifestations; clinical findings (pretreatment). Numerous pink-to-purple facial papulopustules and nodules coalescing into plaques, with crusting and infiltrative hyperkeratotic borders on the face, extending to the frontal hairline with associated alopecia, from the (**A**) frontal and (**B**) right lateral views.

Physical examination revealed many facial firm pink-to-purple papulopustules and nodules coalescing into plaques, with crusting and infiltrative hyperkeratotic borders. These lesions extended into the frontal hairline and scalp with associated alopecia (Fig 1, *A* and *B*). Similar lesions were present on the neck, chest, and ears with significant firm cervical, axillary, and inguinal lymphadenopathy. Mucosal involvement was absent. Complete blood cell count revealed leukocytosis (21.5 × 10^3^/μL) with eosinophilia (36.5%, 9.89 × 10^3^/μL), elevated erythrocyte sedimentation rate to 31 mm/h, C-reactive protein to 16 mg/L, and negative Cryptococcus antigen levels. Tuberculosis, Hepatitis C, and HIV screenings were negative.

**Fig 2:**
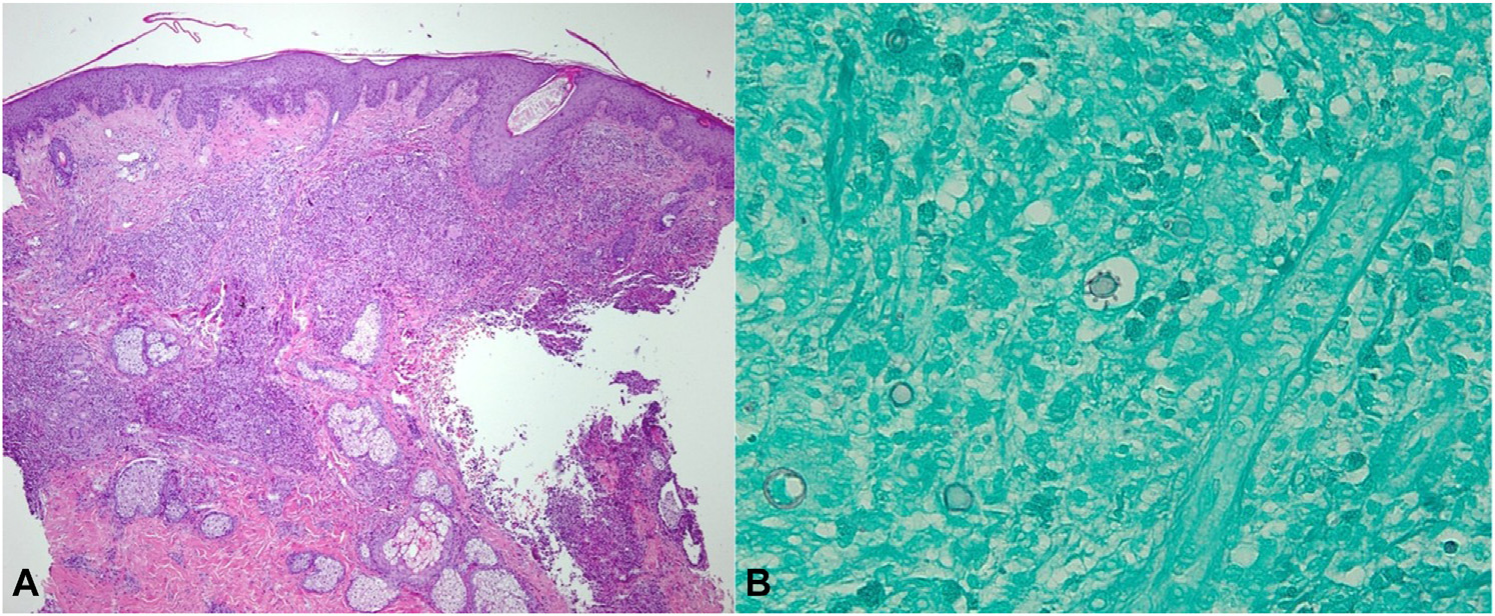
Systemic paracoccidioidomycosis cutaneous manifestations; histologic findings of the skin punch biopsy performed at a lesional site on the face before the administration of systemic antifungal therapy. (**A**) Low-power view hematoxylin and eosin stain (H&E), (**B**) Higher power Grocott methenamine silver (GMS) staining. Large yeasts cells with translucent cell walls with multiple buds, showing the typical “mariners wheel” appearance of paracoccidioidomycosis, with a collection of chronic granulomatous inflammatory cells, and evidence of mild dermal fibrosis.

Contrast-enhanced computed tomography imaging revealed generalized lymphadenopathy in the chest, abdomen, and pelvis with accompanying subcutaneous nodules in the lungs suggestive of pulmonary involvement. Repeat punch biopsy of facial lesions revealed large yeasts cells with translucent cell walls with multiple buds, a collection of chronic granulomatous inflammatory cells, and evidence of mild dermal fibrosis (Fig 2, *A* and *B*).

The patient was admitted and a diagnosis of subacute generalized PCM was rendered. Liposomal amphotericin B 4 mg/kg intravenous daily was given during her hospitalization, and she was discharged on itraconazole 200 mg orally daily for a planned duration of 9 to 12 months.

**Fig 3:**
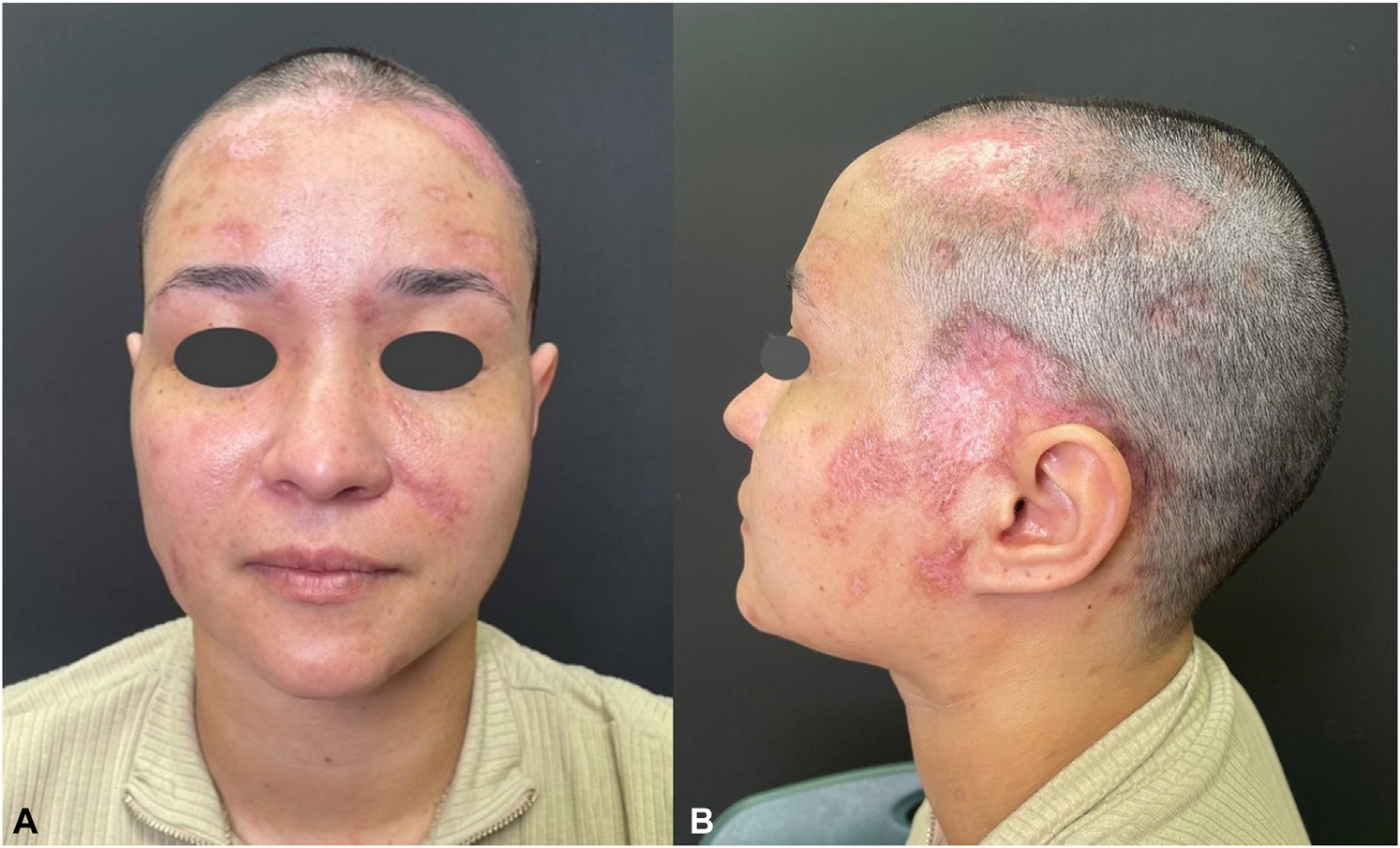
Systemic paracoccidioidomycosis cutaneous manifestations; clinical findings (posttreatment). Multiple atrophic pink plaques with peripheral hyperpigmentation on the face and scalp with appreciable hair regrowth on the previously alopecic surfaces of the scalp, showing resolution of the initial nodular lesions, from the (**A**) frontal and (**B**) left lateral views.

Upon follow-up 3 months later, she had near-complete resolution of her cutaneous lesions and systemic symptoms. Although scarring and dyschromia were appreciable in sites of prior lesions, hair regrowth was present on her scalp, and she endorsed continuous improvement in the appearance of her skin (Fig 3, *A* and *B*).

## Discussion

PCM is the leading cause of death secondary to systemic mycosis in endemic areas, with even higher mortality in immunosuppressed patients.^4^ Mutilating ulcerative lesions affecting the face, nose and oral mucosa, with the latter commonly referred to as “moriform stomatitis,” are the hallmark cutaneous findings in disseminated PCM.^5^ These cutaneous manifestations are often secondary to primary pulmonary disease, which disseminates and can be accompanied by cervical lymphadenopathy. On the other hand, primary cutaneous PCM occurs after direct skin inoculation, often through chewing contaminated food or sticks, and results in the development of verrucous papules/plaques and cutaneous ulceration.^5^ Our patient presented with nodular lesions in the absence of mucosal or nasal involvement. Although diagnostic work-up revealed lung involvement, no immunosuppression was identified, as can be commonly seen with disseminated disease.

Notably, our patient was a woman, as the literature has historically reported that estrogen plays a protective role against PCM infection. Specifically, estrogen is thought to bind PCM proteins and prevent the transition of the saprophytic form to the invasive form of the fungus.^6^ Although a recent publication draws this mechanism of infection inhibition into question, it remains true that clinically evident PCM infections are significantly less common in women, although women who become infected may not have a milder phenotype than men.^7^

A similar presentation of a vegetating case of PCM was reported in a 19-year-old immunocompetent Brazilian woman who presented with violaceous infiltrative plaques with scales and crusting on the face. Her initial biopsy 2 years prior had showed granulomatous infiltrates without fungal elements identified, and it was only when she presented with extensive cutaneous involvement, generalized lymphadenopathy, and systemic symptoms that budding yeast cells were observed on repeat histopathologic examination and the diagnosis of subacute PCM was made.^8^ Lung involvement was absent, as is expected with this rare vegetating subtype of primary cutaneous PCM, characterized by a scarcity of fungal structures accompanying the observed classic granulomatous infiltrates.^9^ Interestingly, in contrast, our patient had significant lung involvement and resultant shortness of breath, which suggests that this vegetating presentation of PCM may not be a skin-limited disease as reported in the current literature,^10^ but may also occur with disseminated PCM with skin infiltration.

Disease progression in our patient because of diagnostic delay may have led to further cutaneous infiltration of fungi, resulting in the yeast forms noted on repeat histopathology examination. This is rather unusual given her lack of identifiable immunosuppression and presumably intact T helper 1 lymphocytic response. It is unclear what prompted the temporary failure of cellular immunity in our patient, leading to a predominantly T helper 2 response and resultant fungal multiplication with disease spread.^10^

In conclusion, this case highlights an atypical presentation of disseminated PCM in an immunocompetent patient. It is important to have a high index of suspicion in patients presenting with infiltrative, vegetating skin lesions and systemic symptoms who have traveled to a PCM endemic region, particularly Brazil, which harbors the highest disease prevalence and is considered hyperendemic.^1^ The absence of budding yeasts alongside granulomatous infiltrates on histopathology should not rule out the diagnosis, but rather, when clinical suspicion remains, special staining should be obtained, such as silver-methenamine stain, to exclude PCM and avoid further disease progression.

## Conflicts of interest

None disclosed.
